# Supplementary material for: Combining multiple functional annotation tools increases coverage of metabolic annotation
Source: BMC Genomics. 2018 Dec 19;19:948. doi: 10.1186/s12864-018-5221-9 (PMC6299973; doi:10.1186/s12864-018-5221-9)
Supplement: Supplementary file 1 — Figure S1. Overlap of annotated genes between the tools (average numbers of genes annotated per genome). Figure S2. Average transporter annotations per genome produced by TransportDB (426.0), KEGG (203.8) and RAST (113.7) and the distributions of their substrate specificities (rank 1 is most specific, rank 5 has no substrate prediction). (DOC 3273 kb) [file 12864_2018_5221_MOESM1_ESM.doc]

Supplementary Figure S1. Overlap of annotated genes between the tools (average numbers of genes annotated per genome).


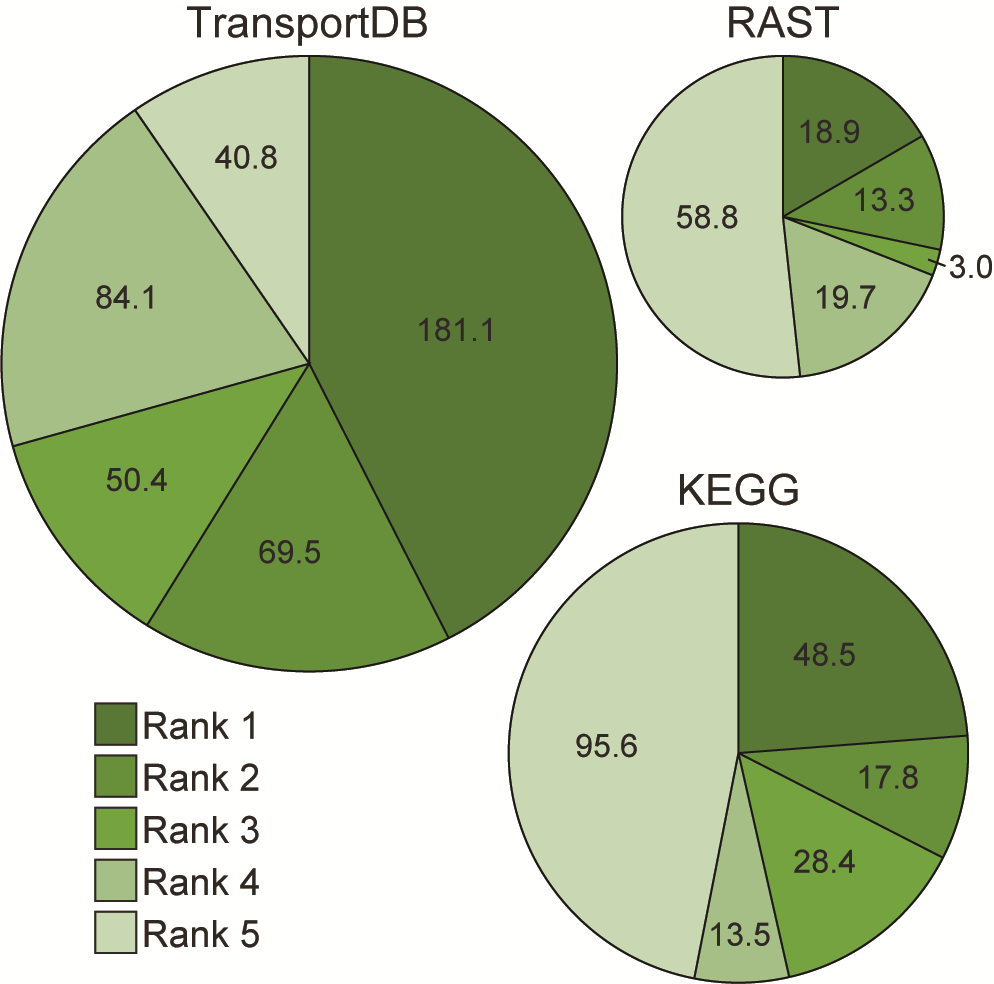


Supplementary Figure S2. Average transporter annotations per genome produced by TransportDB (426.0), KEGG (203.8) and RAST (113.7) and the distributions of their substrate specificities (rank 1 is most specific, rank 5 has no substrate prediction).
